# Supplementary material for: Sustained learned immunosuppression could not prevent local allergic ear swelling in a rat model of contact hypersensitivity
Source: Sci Rep. 2025 Aug 12;15:29456. doi: 10.1038/s41598-025-13850-2 (PMC12340026; doi:10.1038/s41598-025-13850-2)
Supplement: Supplementary file 1 — Supplementary Material 1 [file 41598_2025_13850_MOESM1_ESM.docx]

***Appendix A. Supplementary data***

**Sustained learned immunosuppression could not prevent local allergic ear swelling in a rat model of contact hypersensitivity**

**Authors:**

Yasmin Salem^1^, Stephan Leisengang^1^, Marie Jakobs^1^, Kirsten Dombrowki^1^, Julia Bihorac^1^, Laura Heiss-Lückemann^1^, Sebastian Wenzlaff^1^, Lisa Trautmann^1^, Tim Hagernacker^2^, Manfred Schedlowski^1,3^, Martin Hadamitzky^1^*

**Affiliations:**

^1^Institute of Medical Psychology and Behavioral Immunobiology, Center for Translational Neuro- Behavioral Sciences (C-TNBS), University Hospital Essen, Germany

^2^Department of Neurology, Center for Translational Neuro- Behavioral Sciences (C-TNBS), University Hospital Essen, Germany

^3^Department of Clinical Neuroscience, Osher Center for Integrative Medicine, Karolinska Institute, Stockholm, Sweden

***Corresponding author:**

Martin Hadamitzky, PhD

Institute of Medical Psychology and Behavioral Immunobiology

Center for Translational Neuro- Behavioral Sciences

University Hospital Essen, 45147 Essen, Germany

E-Mail: [martin.hadamitzky@uk](mailto:martin.hadamitzky@uk)-essen.de

**Keywords:** Associative learning, cyclosporine A, contact hypersensitivity, dose reduction, memory-updating


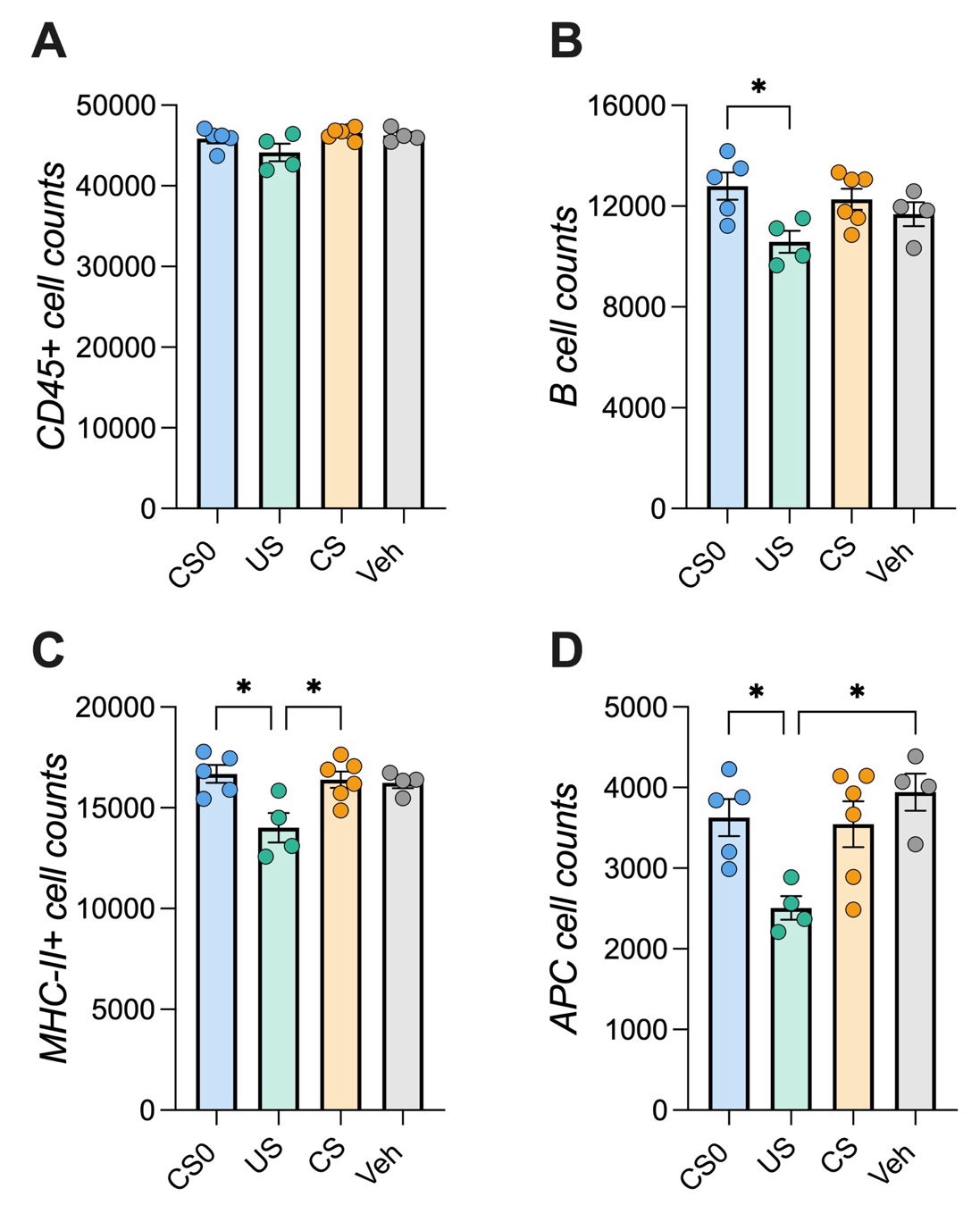


**Supplementary Figure 1. Immune cell subset distribution of draining lymph nodes.** Immune cells of draining lymph nodes (axillary and cervical) were analyzed via flow cytometry. No differences between groups were observed in (**A**) CD45+ cells. A reduction of numbers in the *US* group for (**B**) B-cells (vs *CS0*), (**C**) MHC-II+ (vs *CS0* and *CS*) and (**D**) APCs (vs *CS0* and *Veh*) were shown (ANOVA followed by Bonferroni post hoc analysis, *p<0.05; n=4-6/group). Data are shown as mean ±SEM.
